# Genes involved in muscle contractility and nutrient signaling pathways within celiac disease risk loci show differential mRNA expression

**DOI:** 10.1186/s12881-015-0190-1

**Published:** 2015-06-30

**Authors:** Caroline Montén, Audur H. Gudjonsdottir, Lars Browaldh, Henrik Arnell, Staffan Nilsson, Daniel Agardh, Åsa Torinsson Naluai

**Affiliations:** Diabetes & Celiac Disease Unit, Department of Clinical Sciences, Lund University, Jan Waldenströms gata 35, CRC, 91:10, 202 05 Malmö, Sweden; Department of Pediatrics, Queen Silvia Children’s Hospital, Sahlgrenska Academy, Gothenburg, Sweden; Department of Clinical Science & Education, Karolinska Institute Södersjukhuset, Stockholm, Sweden; Department of Pediatric Gastroenterology, Hepatology & Nutrition, Karolinska University Hospital, Stockholm, Sweden; Department of Mathematical Sciences, Chalmers University of Technology, Gothenburg, Sweden; Department of Medical & Clinical Genetics, Institute of Biomedicine, Sahlgrenska Academy, Gothenburg, Sweden

**Keywords:** Celiac Disease, Gene expression, Small intestinal mucosa, Peripheral blood, Single nucleotide polymorphisms

## Abstract

**Background:**

Risk gene variants for celiac disease, identified in genome-wide linkage and association studies, might influence molecular pathways important for disease development. The aim was to examine expression levels of potential risk genes close to these variants in the small intestine and peripheral blood and also to test if the non-coding variants affect nearby gene expression levels in children with celiac disease.

**Methods:**

Intestinal biopsy and peripheral blood RNA was isolated from 167 children with celiac disease, 61 with potential celiac disease and 174 disease controls. Transcript levels for 88 target genes, selected from celiac disease risk loci, were analyzed in biopsies of a smaller sample subset by qPCR. Differentially expressed genes (3 from the pilot and 8 previously identified) were further validated in the larger sample collection (*n* = 402) of both tissues and correlated to nearby celiac disease risk variants.

**Results:**

All genes were significantly down- or up-regulated in the intestinal mucosa of celiac disease children, *NTS* being most down-regulated (Fold change 3.6, *p* < 0.001). In contrast, *PPP1R12B* isoform C was up-regulated in the celiac disease mucosa (Fold change 1.9, *p* < 0.001). Allele specific expression of *GLS* (rs6741418, *p* = 0.009), *INSR* (rs7254060, *p* = 0.003) and *NCALD* (rs652008, *p* = 0.005) was also detected in the biopsies. Two genes (*APPL2* and *NCALD*) were differentially expressed in peripheral blood but no allele specific expression was observed in this tissue.

**Conclusion:**

The differential expression of *NTS* and *PPP1R12B* indicate a potential role for smooth muscle contractility and cell proliferation in celiac disease, whereas other genes like *GLS*, *NCALD* and *INSR* suggests involvement of nutrient signaling and energy homeostasis in celiac disease pathogenesis. A disturbance in any of these pathways might contribute to development of childhood celiac disease.

**Electronic supplementary material:**

The online version of this article (doi:10.1186/s12881-015-0190-1) contains supplementary material, which is available to authorized users.

## Background

Human leukocyte antigen (HLA) class II molecules on chromosome 6 are necessary factors for celiac disease where 90–95 % of all affected patients carry the HLA-DQA1*05:01-DQB1*02:01 haplotype and the remainder carry either the HLA-DQA1*03:01-DQB1*03:02 or HLA-DQA1*02:01-DQB1*02:02 haplotype [1]. Although the HLA association with celiac disease is well established, 30 % of the general Caucasian population carry risk HLA alleles without developing the disease [2]. Moreover, the odds ratios (OR) for monozygotic twins to be concordant for celiac disease was 17 compared to 1.4 for DQ identical dizygotic twins independent of the DQ at risk genotype [3]. This indicates that non-*HLA* genetic factors play a role in disease susceptibility. Genome wide association studies (GWASs) in celiac disease have identified altogether 58 non-*HLA* risk variants within 40 different loci [4–7]. Expression quantitative trait locus (eQTL) mapping has previously shown that 53 % of celiac disease associated risk regions contain single nucleotide polymorphisms (SNPs) that regulate the expression of nearby genes of which a majority are involved in the control of immune response [5]. With the aim of uncovering additional genetic risk factors for celiac disease, we recently performed a GWAS in Scandinavian sib-pairs with celiac disease [7]. Functional clustering analyses of genes located nearby the strongest associated SNPs generated possible susceptibility genes involved in pathways like polarity and epithelial cell functionality, growth and nutrient signaling, energy homeostasis and intestinal smooth muscle function. In order to reveal a functional role for genes within these pathways in celiac disease, small intestinal mRNA expression for 11 genes were analyzed in a pediatric celiac disease case–control cohort. Eight genes selected were previously found to be differentially expressed in the small intestinal mucosa between celiac disease patients and controls [7]. The remaining three genes were included in a specific functional cluster of cytoplasmic vesicle transport and secretory granules and also expressed differently in the small intestinal mucosa of celiac disease patients and controls in a pilot performed in this study.

The purpose of this study was therefore to confirm the differential gene expression in small intestinal biopsies and peripheral blood using a larger collection of individuals. Furthermore, we aimed to investigate if closely located celiac disease associated SNPs from the sib-pair GWAS inferred risk to disease by affecting biopsy and peripheral blood mRNA expression levels of the eleven differentially expressed genes. Several genes involved in biological pathways of relevance for celiac disease pathogenesis and previously not associated with celiac disease at a transcriptional level were differentially expressed in the small intestine and peripheral blood of our celiac disease case–control cohort. Moreover, some of the genes expression levels were associated with alleles of previous celiac disease associated SNPs. This could indicate a functional role for these genes in triggering and/or upholding the disease.

## Methods

### Study subjects and diagnostic criteria

Biopsies from duodenum and peripheral blood samples were consecutively collected from 402 children (167 with celiac disease at median 7.1 years of age, 107 girls and 60 boys; 61 with potential celiac disease at median 8.8 years of age, 42 girls and 19 boys; 174 controls at median 11.9 years of age, 97 girls and 77 boys) referred to upper endoscopy at four Swedish pediatric gastroenterology units located at Scania University hospital in Malmoe, Södersjukhuset in Stockholm, Karolinska University hospital in Stockholm and Queen Silvia Children’s hospital in Gothenburg. All participants were informed about the aim of the study and a parental written consent was obtained for each child. The ethical committee at Gothenburg University approved the study.

In order to avoid variations in grading of the biopsies by different pathologists in this multicenter study, biopsies from all the patients were in retrospect blindly classified by the same pathologist. Due to the commonly observed atrophic patchiness of the duodenum in celiac disease patients, classification was determined from a biopsy in the same part of duodenum as the biopsy used for gene expression analyses. For the purposes of this study, celiac disease patients were defined as being positive for tissue transglutaminase (tTG) autoantibodies in blood plasma as previously described [8] and a biopsy showing characteristic villous atrophy of the distal part of duodenum. Among the children with celiac disease, 6 had a Marsh score 2, 31 had a Marsh score 3a, 63 had a Marsh score 3b and 67 a Marsh score 3c. Potential celiac disease was defined as being tTG autoantibody positive and having a normal biopsy. Children being tTG autoantibody negative with a normal biopsy were included as disease controls.

### Sample preparation

The intestinal biopsy was immediately after collection put in the RNA stabilizing reagent RNA*later* (Life Technologies, CA, US) and frozen in −80 °C until RNA extraction was carried out using the miRNeasy Mini Kit (Qiagen, Germany) or the Maxwell® 16 Total RNA Purification Kit (Promega). The RNA quality and quantity was checked with a NanoDrop 2000 spectrophotometer and a 2100 Bioanalyzer (Agilent Technologies).

Peripheral blood samples were also collected from each patient, one in RNA stabilizing reagent (Tempus™), one in lithium heparin and one in EDTA. DNA was extracted from EDTA stabilized whole blood using the Maxwell® 16 LoDNA Purification Kit (Promega). RNA was isolated from the Tempus™ tubes using the Tempus™ Spin RNA Isolation kit (Life Technologies, CA, US) or the Maxwell® 16 Total RNA Purification Kit (Promega). A DNase treatment with AbsoluteRNA was included in the Tempus™ RNA extraction protocol. Complimentary DNA (cDNA) was synthesized from total RNA using the SuperScript VILO cDNA kit according to the manufacturers protocol (Life Technologies, CA, US).

### Selection criteria for candidate genes

The 603 SNPs used to identify genes for the expression analyses had previously been nominally associated with celiac disease in our family GWAS [7]. The SNPs were chosen based on three different inclusion criteria 1) *p*-value < 3.0*10^−4^ in the transmission disequilibrium test (TDT) 2) OR of <0.2 or >5 combined with *p* < 1.5*10^−3^ 3) Combined *p* < 5.0*10^−5^ with the Dubois et al. population based GWAS [5]. Two different selection procedures were used to choose the genes tested for mRNA expression in small intestinal biopsies (Additional file 1: Figure S1: Selection procedure for target genes). The first selection included 8 genes previously found to be differentially expressed in the small intestine between celiac disease patients and controls [7]. The second set included 54 genes from the most significant functional annotation cluster (Additional file 2: Table S1: cytoplasmic vesicle transport and secretory granules) generated by DAVID (http://david.abcc.ncifcrf.gov/) as well as an additional 34 functionally related genes.

### Gene expression analyses and genotyping

In the following procedure, mRNA expression for all 96 selected target genes was measured in the small intestinal mucosa using TaqMan technology. Eight genes (*ADCY9: Adenylate cyclase 9, APPL2: Adaptor protein, phosphotyrosine interaction, PH domain and leucine zipper containing 2, GLS: Glutaminase, INSR: Insulin receptor, KIF13A: Kinesin family member 13A, PDK1: Pyruvate dehydrogenase kinase isozyme 1, PPP1R12B* transcript NM032103.2*: Myosin light chain phosphatase catalytic subunit 12B, PRR5L: Proline rich 5 like*) in the previous set of 35 target genes were shown to be differentially expressed in the small intestinal mucosa from a subset of 89 individuals within our celiac disease case–control cohort in the paper by Östensson et al. [7]. The second set of 88 target genes and 8 reference genes were analyzed in this study using a subset of 80 individuals and 384 well TaqMan low density arrays (Life Technologies, CA, US). For each individual and 96 gene set, 0.5 μg cDNA was loaded. Three genes (*HSPB1: Heat shock 27 kDa protein 1, NCALD: Neurocalcin* δ*, NTS: Neurotensin*) from this pilot experiment were differentially expressed between celiac disease patients and controls after correction for multiple testing (Additional file 3: Table S2).

Gene expression for the 11 selected genes was further analyzed in the small intestinal mucosa and in peripheral blood from all 402 children in the celiac disease case–control cohort. The TaqMan qPCR reaction was run in duplicates and the samples were distributed randomly on the plates with a final cDNA concentration of 1 ng/μl. The raw data was analyzed using SDS version 2.4 and RQ manager 1.2.1. Samples with a Ct replicate difference of more than one cycle were excluded from analysis. Transcript quantification was determined using the ΔΔCt method [9] where each target gene was normalized to an arithmetic mean of the two reference genes *TRAP1* (Hs00972326_m1) and *ACTB* (Hs00357333_g1). Evaluation of reference gene stability in both tissues was performed with the geNorm module in the qBase^PLUS^ v2.1 (Biogazelle, Belgium). Genotyping of SNPs was performed with KASP-technology (KBioscience/LGC Genomics) and KASP-on-demand assays. All qPCR reactions and genotyping was run on a 7900HT Sequence Detection System (Life Technologies, CA, US).

### Statistical analyses

Gene expression levels in intestinal mucosa and peripheral blood were compared between groups using deltaCt-values (i.e., log expression levels) as outcome in linear model with age and sex as covariates. The allele specific gene expression analysis was conducted in the celiac disease group and control or potential celiac disease group separately using linear regression with allele count as predictor and adjusted for age and sex. A logistic regression model was used to associate allele count with celiac disease outcome (potential celiac disease merged with controls) and with subjects of non-Caucasian origin excluded. Spearman rank test was used to analyze the correlations in gene expression between tissues. A *p*-value <0.05 was considered significant (Bonferroni-Holm procedure was used to correct for multiple testing [10]). All statistical analyses were conducted in R or SPSS version 21 for Windows (SPSS, Chicago, IL, USA).

## Results

In the pilot gene expression experiment of 88 target genes in the small intestinal mucosa, only 44 genes (50 %) were expressed by at least 50 % of the patients (Additional file 4: Table S3) and three genes (*NTS*, *NCALD*, *HSPB1*) showed a significant differential expression between cases and controls after correction for multiple testing (Additional file 3: Table S2).

**Fig. 1:**
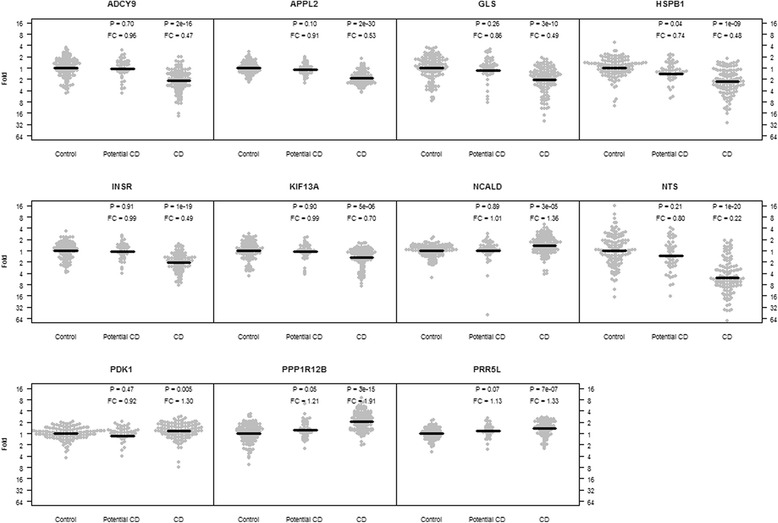
Gene expression in small intestinal mucosa vs. celiac disease status. Fold change and p-value are presented for the celiac disease group (*n* = 167) and the potential celiac disease group (*n* = 61) with the control group (*n* = 174) set as reference and adjusting for age and sex

When including additional patients and controls we could not detect any significant difference in gene expression between potential celiac disease patients and controls in neither peripheral blood nor in the intestinal biopsies (Fig. 1, blood data not shown). These two groups were therefore combined and compared to biopsy confirmed celiac disease patients. In an analysis including only new cases and controls, all the 11 selected target genes showed a significant differential expression in the small intestinal epithelium (Table 1). Seven genes were down-regulated in the biopsies of celiac disease patients, among them *NTS* down-regulated 3.6 fold and the *INSR*, *APPL2*, *ADCY9*, *GLS*, *HSPB1* and *KIF13A* 1.5-2.2 fold in the patient group (p < 0.001). On the contrary, four genes including the specific isoform C of *PPP1R12B, PRR5L*, *PDK1* and *NCALD* were up-regulated in the celiac small intestinal mucosa (*PPP1R12B* fold change = 1.9; *PRR5L*, *PDK1* and *NCALD* fold change = 1.2-1.4). Biopsy expression levels for all 11 genes significantly correlated to the degree of lesion in the small intestine as well as to plasma tTG autoantibody levels (data not shown). In peripheral blood, disease status affected the expression of the *APPL2* and *NCALD* genes (Table 1). The gene *APPL2* was down-regulated and *NCALD* up-regulated in both peripheral blood and the small intestine of celiac disease patients. The expression levels correlated significantly between the two types of tissue (APPL2: *r* = 0.15, *p* = 0.01; NCALD: *r* = 0.18, *p* = 0.002). The *PPP1R12B* isoform C and *NTS* gene transcript was not expressed in peripheral blood and the *ADCY9, HSPB1* and *PDK1* genes were excluded from analysis in peripheral blood due to problems with transcript detection. No gene expression levels in blood correlated with plasma tTG autoantibodies (data not shown).

**Table 1:** a) Small intestinal biopsy and b) Peripheral blood gene expression vs. celiac disease status. Fold change and effect direction is presented for the celiac disease group (*n* = 167) with the control and potential celiac disease group (*n* = 235) set as reference. Individuals in the pilot data sets were excluded from the biopsy gene expression analysis. The analysis was adjusted for age and sex in both tissues

a. Small intestinal biopsy			
Gene symbol	Gene name	Fold (95 % CI) change	p_c_
ADCY9	Adenylate cyclase 9	2.2 (1.8-2.7) DOWN	1.3E-13
APPL2	Adaptor protein, phosphotyrosine interaction, PH domain and leucine zipper containing 2	1.9 (1.7-2.1) DOWN	1.9E-25
GLS^a^	Glutaminase	2.0 (1.6-2.5) DOWN	1.1E-08
HSPB1	Heat shock 27 kDa protein 1	1.9 (1.5-2.3) DOWN	5.9E-08
INSR	Insulin receptor	2.1 (1.8-2.5) DOWN	3.0E-18
KIF13A	Kinesin family member 13A	1.5 (1.3-1.7) DOWN	3.6E-05
NCALD	Neurocalcin delta	1.4 (1.2-1.6) UP	1.7E-04
NTS	Neurotensin	3.6 (2.7-4.9) DOWN	5.5E-15
PDK1	Pyruvate dehydrogenase kinase, isozyme 1	1.3 (1.1-1.6) UP	7.5E-03
PPP1R12B^a^	Myosin light chain phosphatase catalytic subunit 12B	1.9 (1.6-2.2) UP	2.5E-12
PRR5L	Proline rich 5 like	1.2 (1.1-1.4) UP	3.6E-03
b. Peripheral blood			
Gene symbol	Gene name	Fold (95 % CI) change	p (p_c_)
ADCY9	Adenylate cyclase 9	Low transcript quality	Low transcript quality
APPL2	Adaptor protein, phosphotyrosine interaction, PH domain and leucine zipper containing 2	1.3 (1.1-1.5) DOWN	0.003 (0.02)
GLS^a^	Glutaminase	1.1 (0.8-1.4) DOWN	0.63
HSPB1	Heat shock 27 kDa protein 1	Low transcript quality	Low transcript quality
INSR	Insulin receptor	1.2 (1.0-1.4) DOWN	0.088
KIF13A	Kinesin family member 13A	1.3 (1.0-1.8) UP	0.10
NCALD	Neurocalcin delta	1.5 (1.1-2.0) UP	0.008 (0.04)
NTS	Neurotensin	Not expressed	Not expressed
PDK1	Pyruvate dehydrogenase kinase, isozyme 1	Low transcript quality	Low transcript quality
PPP1R12B^a^	Myosin light chain phosphatase catalytic subunit 12B	Not expressed	Not expressed
PRR5L	Proline rich 5 like	1.3 (1.0-1.8) UP	0.081

^a^Gene located in celiac disease genome-wide significant region

**Fig. 2:**
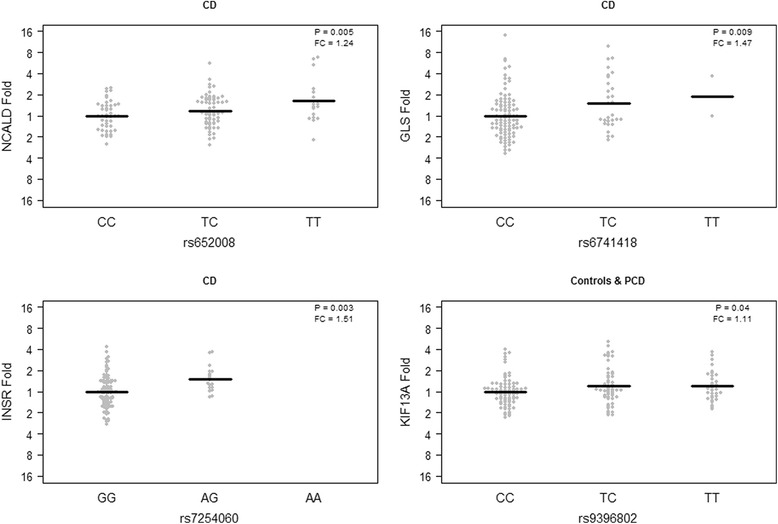
Allele specific gene expression in the small intestinal mucosa. The analysis was performed in celiac cases and controls separately using linear regression adjusting for age and sex to correlate expression levels to minor allele count

Four genes (*GLS, INSR, KIF13A* and *NCALD*) showed allele specific gene expression in the small intestinal mucosa that was dependent on the disease state of the patient (Fig. 2) (Table 2). In biopsies from celiac disease patients, minor allele carriers of the INSR rs7254060 SNP showed decreased INSR gene expression levels (INSR rs7254060; *p* = 0.003) and minor GLS rs6741418 carriers showed decreased GLS gene expression levels (GLS rs6741418; *p* = 0.009). The NCALD gene expression in biopsies was decreased in celiac disease patients carrying the minor allele of SNP rs652008 (*p* = 0.005). We could not detect any allele specific gene expression in peripheral blood (Table 2).

**Table 2:** a) Small intestinal biopsy or b) Peripheral blood gene expression levels vs SNP. The analysis was stratified according to disease status and performed in the celiac disease group (*n* = 167) and control or potential celiac disease group (*n* = 235) separately

			Controls & PCD		Celiac disease	
a. Small intestinal biopsy						
Gene symbol	SNP ID	Alleles^a^	Fold change	p	Fold change	p (p_c_)
ADCY9^b^	rs882820	G:**A**	1.04 UP	0.67	1.21 DOWN	0.08
APPL2	rs10861406	G:**A**	1.01 DOWN	0.88	1.01 DOWN	0.79
GLS^b,c^	rs6741418	C:**T**	1.04 UP	0.71	1.47 DOWN	0.009
HSPB1	rs1019096	G:**A**	1.03 DOWN	0.77	1.05 DOWN	0.66
INSR^b^	rs7254060	G:**A**	1.05 DOWN	0.61	1.51 DOWN	**0.003 (0.03)**
KIF13A	rs9396802	C:**T**	1.11 DOWN	0.04	1.09 DOWN	0.22
NCALD^b^	rs652008	C:**T**	1.00 DOWN	0.97	1.24 DOWN	**0.005 (0.05)**
NTS^b^	rs11104365	C:**T**	1.02 UP	0.85	1.17 DOWN	0.33
PDK1^b^	rs4972810	G:**A**	1.03 DOWN	0.85	1.09 DOWN	0.71
PPP1R12B^c^	rs12734338	T:**C**	1.02 UP	0.95	1.04 UP	0.42
PRR5L	rs10501156	C:**A**	1.07 DOWN	0.29	1.62 DOWN	0.92
b. Peripheral blood						
Gene symbol	SNP ID	Alleles^a^	Fold change	p	Fold change	p
ADCY9^b^	rs882820	G:**A**	Low transcript quality		Low transcript quality	
APPL2	rs10861406	G:**A**	1.02 UP	0.85	1.00 UP	0.97
GLS^b,c^	rs6741418	C:**T**	1.23 DOWN	0.18	1.31 DOWN	0.15
HSPB1	rs1019096	G:**A**	Low transcript quality		Low transcript quality	
INSR^b^	rs7254060	G:**A**	1.10 UP	0.45	1.18 DOWN	0.43
KIF13A	rs9396802	C:**T**	1.07 DOWN	0.59	1.01 DOWN	0.97
NCALD^b^	rs652008	C:**T**	1.15 UP	0.29	1.03 DOWN	0.85
NTS^b^	rs11104365	C:**T**	Not expressed		Not expressed	
PDK1^b^	rs4972810	G:**A**	Low transcript quality		Low transcript quality	
PPP1R12B^c^	rs12734338	T:**C**	Not expressed		Not expressed	
PRR5L	rs10501156	C:**A**	1.22 DOWN	0.46	1.15 DOWN	0.64

The p-values represent the association between gene expression and minor allele count using a linear regression model corrected for age and sex

Significant associations are shown in bold type

^a^Minor allele in bold type

^b^Gene with an eQTL effect in peripheral blood according to the Blood eQTL browser

^c^Gene located in celiac disease genome-wide significant region

In the analyses comparing SNP alleles with celiac disease status (data not shown), the *PRR5L* intronic SNP rs10501156 was associated with celiac disease. Minor allele carriers of this SNP showed a decreased risk for celiac disease compared to controls and potential celiac disease patients (OR = 0.42; *p* = 0.007).

## Discussion

In the present study we aimed to elucidate if non-coding SNPs, previously associated with celiac disease in our GWAS, could regulate nearby gene expression in the small intestinal mucosa and in peripheral blood. Our major finding was that four SNPs affected gene expression levels in the small intestinal mucosa and this was dependent on celiac disease activity. All the eleven investigated genes included those located close to the four suggested eQTL SNPs, showed a differential biopsy expression between children with celiac disease and those without the disease. We could not detect any allele specific expression in peripheral blood from the same individuals. However, two genes were differentially expressed in peripheral blood between celiac disease patients and controls and the direction of the change was the same as in the small intestinal mucosa and each gene correlated significantly between the two tissues.

Two of our eleven selected genes (GLS and PPP1R12B) map within celiac disease genome-wide significant loci. Even though most of the selected SNPs that are located close to the eleven genes are not genome-wide significant, they could still be of interest when trying to understand disease mechanisms. The genes that were selected for this study are involved in epithelial cell functionality, smooth muscle contractility and nutrient signaling pathways, all of biological relevance for celiac disease pathogenesis. Transcript levels for nearly all of these genes have previously been analyzed in celiac disease small intestinal mucosa [11]. However, none of the genes have to our knowledge been previously associated with the disease at a transcriptional level or with celiac disease risk SNPs. Altered expression in small intestinal celiac disease mucosa have previously been observed for other genes involved in similar pathways like cell proliferation and differentiation, apoptosis, transport and metabolism [11, 12]. In one such study, a majority of the genes involved in cell proliferation and differentiation pathways showed a decreased gene expression in biopsy confirmed celiac disease patients [11]. Most of the genes in our expression analysis were likewise down-regulated in the small intestine of celiac disease patients compared with controls. The *NTS* transcript was down-regulated 3.6 fold in celiac disease mucosa but not detected in peripheral blood. The NTS encoded neuropeptide is mainly expressed in the central nervous system but also act as a hormone in the gastrointestinal tract where its main function is to control smooth muscle contractions stimulated by intake of fat [13]. The role of NTS as a protector of intestinal atrophy by reducing the rate of apoptosis in intestinal crypts was recently suggested [14]. The NTS peptide has also been shown to stimulate epithelial restitution following a chronic intestinal inflammation in mouse colon through up-regulation of a COX-2 dependent pathway [15]. Renewal of epithelial cells in the gut is a continuous process dependent on a proper balance between proliferation and apoptosis. Increased apoptosis and crypt cell proliferation has previously been observed in active celiac disease [16]. Altered expression of genes in the control of this balance could lead to crypt hyperplasia and eventually to villous atrophy.

We observed a tissue specific expression for the *PPP1R12B* isoform C, which was up-regulated in celiac disease intestinal mucosa albeit not expressed in peripheral blood. The PPP1R12B protein is a phosphatase that in similarity to NTS control muscle contractility and when activated causes muscle relaxation [17]. The specific *PPP1R12B* isoform encodes a 208 amino acid long C-terminal part of the protein (called small subunit M20) that interacts with the interleukin-16 (IL-16) precursor protein pro-IL-16 [18, 19]. Mature IL-16 function as a chemo attractant factor for CD4^+^ T-cells and also regulate HLA class II expression and release of other pro-inflammatory cytokines [20]. Although its effect of interaction with PPP1R12B is currently unknown, it indicates a connection between changes in cytoskeleton rearrangements and the immune response predisposing to the chronic inflammation in celiac disease.

In the current study, we focused on SNPs in cis position of target genes since previously other celiac disease associated SNPs were shown to affect nearby gene expression levels [5]. SNPs in a cis position could alter gene expression levels, timing and localization by altering binding sites for transcription regulatory proteins [21]. Several of the SNPs affecting gene expression levels in our study are known to act as binding sites for transcription factors (rs6741418-EP300, rs7254060-NFKB1, rs652008-GATA5-6) and are therefore likely to have a functional effect [22]. A nominally significant effect of our analyzed SNP rs6741418 on *GLS* gene expression levels in the small intestinal mucosa was observed in our celiac disease patients. This SNP is located in one of the strongest GWAS celiac disease risk loci about 22 kb upstream of the *GLS* gene. A previous *GLS* promoter analysis identified several binding sites for the STAT1 transcription factor and revealed that cytokine (IFN-α) mediated activation of the *GLS* promoter requires STAT1 phosphorylation [23]. In our patients, *GLS* gene expression was significantly decreased 2-fold compared to controls and a decreased GLS activity has previously been observed in the intestine of untreated celiac disease patients [24]. The main function of GLS is to convert glutamine to glutamate and ammonia, an important process to maintain energy balance in the gut.

Other studies have investigated and some also found allele specific gene expression in the small intestinal mucosa in celiac disease although performed in very small sample sizes [25, 26]. Important factors to consider when studying small changes in gene expression caused by non-coding SNPs in complex diseases are the use of relevant tissue or cell type under the right conditions and use of sufficiently large datasets. Several eQTL databases include more easily accessible peripheral blood samples or specific isolated blood cell populations, the largest being the blood eQTL database [27] and a monocyte dataset of 1490 unrelated individuals [28]. Comparing our data with these databases we found that six of our analyzed genes (GLS, NCALD, INSR, ADCY9, NTS, PDK1) previously have shown significant eQTL effect in peripheral blood. In our study, three of these six genes (GLS, NCALD, INSR) showed a significant eQTL effect and were also differentially expressed in the small intestine. Our NCALD eQTL SNP is in linkage disequilibrium (LD) with two nominally significant NCALD eQTL SNPs in the database. The remaining genes in the database were in eQTL with other SNPs than our selected one. Two SNPs that failed to show an allele specific effect in our study has been shown to affect the expression of another closely located gene (rs10501156-*TRAF6* [29], rs1019096-*ZP3* [28, 30]) and two of our suggested eQTL SNPs (rs6741418/*GLS* and rs7254060/*INSR*) are in LD with another SNP that is associated with a specific phenotype (rs6741418/rs10497709: fasting triglyceride levels [31], rs6741418/rs3024896: fasting insulin levels [32], rs7254060/rs2115386: diabetic retinopathy [33]). Other SNPs have been shown to affect expression levels for some of our target genes in brain tissue (*PRR5L*), liver (*INSR*, *ADCY9*) and lymphoblastoids (*APPL2*) according to the NCBI eQTL browser.

A major advantage with our study is the large collection of biopsies from over 400 individuals which increases our power to detect differences in gene expression especially against variants with lower allele frequency. Another advantage is that the diagnosis of our patients is determined from a biopsy in the same part of duodenum as the biopsy used for gene expression analyses. This is important in order to avoid biased gene expression results due to the commonly observed patchy inflammation in the celiac disease small intestinal mucosa. A possible problem that could give a bias to the observed differential expression in our study is the heterogeneous cell population in the biopsies. However, a majority of the selected genes in this study are not known to be preferentially expressed by specific cell types that could have been lost due to villous atrophy.

## Conclusion

We have shown that some of our celiac disease associated SNPs from our published family GWAS affect transcription in the small intestinal epithelium of genes not previously implicated in the disease. The differential expression of *NTS* and *PPP1R12B* indicate a potential role for smooth muscle contractility and cell proliferation, whereas other genes like *GLS*, *NCALD* and *INSR* suggests involvement of nutrient signaling and energy homeostasis in celiac disease pathogenesis. Individuals being genetically susceptible for the effect of gluten on these pathways might be more prone to trigger the immune system and develop celiac disease. The proposed functional role of the genes and variants in celiac disease should be further addressed at post-translational level and in specific disease cell populations.

## Additional files

Additional file 1: Figure S1. Selection procedure for candidate genes. Two different procedures used for selection of candidate genes for pilot expression analysis in small intestinal biopsies. ^1^Ostensson M et al., *PloS one* 2013, 8 (8). Additional file 2: Table S1. The top three functional annotation cluster generated in DAVID. Genes from cluster 1 were chosen for gene expression analyses in small intestinal biopsies from our celiac disease case control cohort. Additional file 3: Table S2. Expressed genes in pilot analysis of 88 genes in the small intestinal biopsies from 80 children. Genes from the most significant DAVID annotation cluster and associated pathways were selected. Transcript levels were compared to celiac disease status (42 celiac disease patients, 38 disease controls) using two-sample *t*-test and significant associations (*p* < 0.05) after Bonferroni correction are shown in bold. Additional file 4: Table S3. Unexpressed genes in pilot analysis of 88 genes in the small intestinal biopsies from 80 children. Modestly expressed genes (>50 % of the children with transcript levels Ct > 40) were excluded from further analysis.

## Abbreviations

SNP: Single nucleotide polymorphism
HLA: Human leukocyte antigen
GWAS: Genome wide association studies
eQTL: Expression quantitative trait locus
tTG: Tissue transglutaminase
TDT: Transmission disequilibrium test
