# Supplementary figures and images for: Genes involved in muscle contractility and nutrient signaling pathways within celiac disease risk loci show differential mRNA expression
Source: BMC Med Genet. 2015 Jun 30;16:44. doi: 10.1186/s12881-015-0190-1 (PMC4630939; doi:10.1186/s12881-015-0190-1)

### Target gene selection 1

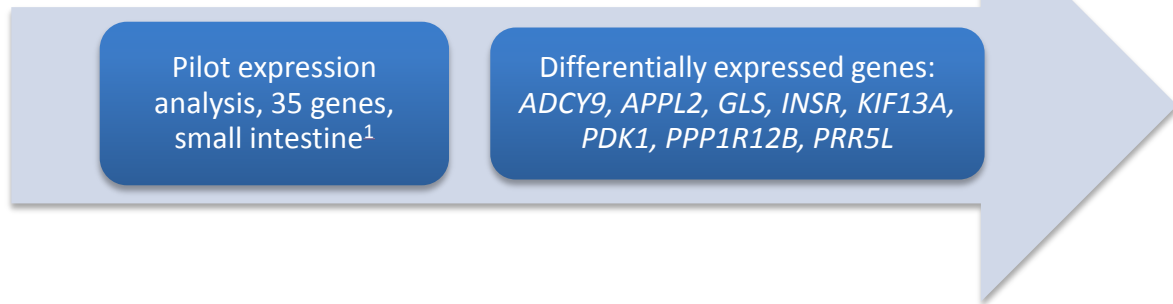

### Target gene selection 2

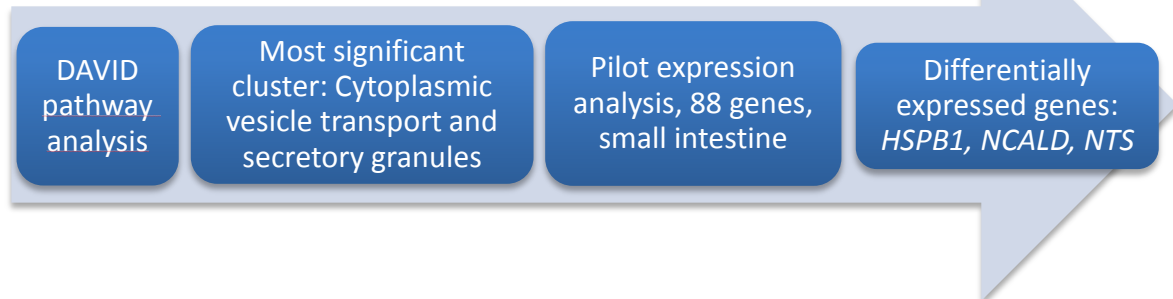

Supplement: Additional file 1: Figure S1. — Selection procedure for candidate genes. Two different procedures used for selection of candidate genes for pilot expression analysis in small intestinal biopsies. 1Ostensson M et al., PloS one 2013, 8 (8). [file 12881_2015_190_MOESM1_ESM.pdf]
